# Multimethod Evaluation of the Novel Reciproc Minima System: Geometric Design, Mechanical Performance, and Irrigation Dynamics

**DOI:** 10.3390/dj14080471

**Published:** 2026-08-02

**Authors:** Emmanuel J. N. L. Silva, Jorge N. R. Martins, Victor T. L. Vieira, Mário Rito Pereira, Ricardo Pinto, Murilo P. Alcalde, Marco A. H. Duarte, Duarte Marques, Marco A. Versiani

**Affiliations:** 1 Department of Endodontics, Grande Rio University (UNIGRANRIO), Rio de Janeiro 21210-623, RJ, Brazil; 2 Department of Endodontics, Rio de Janeiro State University (UERJ), Rio de Janeiro 20550-013, RJ, Brazil; 3 Faculdade de Medicina Dentária, Universidade de Lisboa, 1600-277 Lisboa, Portugal; 4 Grupo de Investigação em Bioquimica e Biologia Oral, Unidade de Investigação em Ciências Orais e Biomédicas (UICOB), Faculdade de Medicina Dentária, Universidade de Lisboa, 1600-277 Lisboa, Portugal; 5 Centro de Estudo de Medicina Dentária Baseada na Evidência (CEMDBE)—Cochrane Portugal, Faculdade de Medicina Dentária, Universidade de Lisboa, 1600-277 Lisboa, Portugal; 6 Laboratory for Instrumentation, Biomedical Engineering and Radiation Physics (LIBPhys-FCT UID/FIS/04559/2013), Faculdade de Medicina Dentária, Universidade de Lisboa, 1600-277 Lisboa, Portugal; 7 Egas Moniz Center for Interdisciplinary Research (CiiEM), Egas Moniz School of Health & Science, 2829-511 Caparica, Portugal; 8 Department of Endodontics, São Paulo University (USP), Bauru 17012-901, SP, Brazil; 9 Oral Health Center, Brazilian Military Police, Belo Horizonte 30350-190, MG, Brazil

**Keywords:** computational fluid dynamics, endodontics, NiTi, mechanical tests, reciprocating instruments

## Abstract

**Objectives:** To compare the geometric design, metallurgical properties, mechanical performance, and irrigation dynamics of Reciproc Minima (M20 and M25) and Reciproc Blue R25 instruments. **Methods:** One hundred and eighty instruments (n = 60/group) were evaluated. Geometry was analyzed using stereomicroscopy, scanning electron microscopy, and 3D surface scanning. Metallurgical characteristics were assessed by energy-dispersive X-ray spectroscopy and differential scanning calorimetry. Mechanical performance tests (n = 10/group) included cyclic fatigue, torsional resistance, bending resistance, buckling resistance, and cutting efficiency. Irrigation dynamics were examined through computational fluid dynamics simulations based on a micro-CT-derived mandibular molar model prepared according to each system and combined with open-ended, side-vented, or double side-vented needles. Data were analyzed using one-way ANOVA or Kruskal–Wallis tests (α = 0.05). **Results:** The results showed that blade dimensions increased progressively from Minima M20 to Reciproc Blue R25. All instruments had S-shaped cross-sections and non-active tips. Energy-dispersive spectroscopy confirmed near-equiatomic NiTi composition, and similar phase transformation temperatures were observed across groups. Minima M20 showed the highest cyclic fatigue resistance (*p* < 0.0001), whereas Minima R25 exhibited greater angular deflection (*p* < 0.0001). Reciproc Blue R25 had the highest buckling resistance and lowest flexibility (*p* < 0.0001). M25 showed the lowest axial force, indicating the numerically highest cutting efficiency, but it did not differ significantly from Reciproc Blue R25 (*p* > 0.05). No needle delivered irrigant to working length. The open-ended needle achieved greater apical penetration, particularly with Reciproc Blue R25. Minima M20 generated the highest wall shear stress, and Reciproc Blue R25 the lowest apical pressure. **Conclusions:** Reciproc Minima and Reciproc Blue R25 showed similar metallurgical characteristics; however, differences in geometric design resulted in distinct mechanical behaviors and irrigation fluid dynamics. These findings suggest that low-taper reciprocating instruments may represent a conservative alternative in anatomically challenging canals, while clinicians should consider the associated differences in mechanical behavior and irrigation dynamics when selecting the most appropriate instrument.

## 1. Introduction

Root canal preparation critically influences irrigation efficacy, disinfection, obturation quality, and long-term tooth survival [1,2]. Contemporary endodontics increasingly advocates minimally invasive strategies that preserve radicular and pericervical dentin, particularly in anatomically complex roots [3]. Within this framework, the geometric design and metallurgical properties of nickel–titanium (NiTi) instruments, such as taper, core diameter, cross-sectional configuration, and heat treatment, directly affect shaping behavior, mechanical safety, and clinical performance [4].

Reciprocating NiTi systems have gained widespread acceptance due to their simplified protocols and improved resistance to cyclic fatigue [5,6]. The Reciproc family introduced in 2011 a single-file concept with the original Reciproc M-Wire (VDW GmbH, Munich, Germany), later enhanced by Reciproc Blue [7] through proprietary heat treatment that increased flexibility while maintaining the same kinematics and general design [5,8]. More recently, Reciproc Minima was developed as a low-taper derivative of the Reciproc Blue platform. While preserving alloy composition and reciprocating motion, it incorporates reduced core dimensions and a more regressive taper, aiming to promote conservative shaping and improved negotiation of narrow or curved canals. However, taper reduction may influence flexibility, torsional behavior, buckling resistance, and cutting efficiency. Instrument geometry can also affect irrigant flow patterns, wall shear stress, and apical pressure [1], which are clinically relevant for biofilm disruption and extrusion risk [9]. Because direct experimental assessment of irrigant flow within complex root canal systems is technically challenging, computational fluid dynamics (CFD) has emerged as a reliable and reproducible method for quantifying irrigation parameters that cannot be directly measured, including flow distribution, wall shear stress, and apical pressure. Consequently, combining CFD with conventional mechanical testing enables a more comprehensive evaluation of instrument performance by evaluating not only its mechanical behavior but also its potential influence on irrigation effectiveness. Despite the growing emphasis on conservative shaping, the mechanical and fluid-dynamic implications of low-taper reciprocating instruments remain insufficiently characterized.

Although previous investigations have evaluated isolated mechanical properties or shaping performance of reciprocating NiTi instruments [10], evidence regarding newly introduced low-taper reciprocating systems remains scarce. Moreover, previous studies have typically focused on a single aspect of instrument performance, whereas no study has simultaneously integrated detailed geometric characterization, metallurgical analysis, comprehensive mechanical testing, and computational fluid dynamics to determine how taper reduction influences both instrument behavior and irrigation performance. This multimethod approach provides a more complete understanding of the potential benefits and trade-offs associated with conservative reciprocating instrumentation.

Therefore, the aim of this study was to evaluate the influence of the geometric differences in the mechanical performance and irrigation dynamics of the Reciproc Minima system (M20 and M25) compared with Reciproc Blue (R25 Blue). Reciproc Blue R25 was selected as the reference instrument because it represents the established reciprocating platform from which Reciproc Minima was derived, allowing the influence of the overall geometric differences between the instruments to be investigated while minimizing confounding factors related to alloy composition and reciprocating kinematics. The null hypothesis was that no significant differences exist between Reciproc Minima and Reciproc Blue in terms of their geometric features, mechanical properties, or irrigation dynamics.

## 2. Materials and Methods

### 2.1. Instrument Selection

A total of 180 NiTi reciprocating instruments (n = 60 per group) with 0.20 mm (M20; VDW GmbH, Munich, Germany) or 0.25 mm tip sizes (M25 and R25 Blue; VDW GmbH, Munich, Germany), variable taper, and a standardized length of 25 mm were included in this study and analyzed/tested only once. All instruments were examined under ×13.6 magnification (Opmi Pico microscope; Carl Zeiss Surgical, Oberkochen, Germany), and no structural irregularities or manufacturing defects were detected, allowing all instruments to be included in the analysis.

### 2.2. Geometric Design

Instrument design was assessed using stereomicroscopy (n = 3), scanning electron microscopy (SEM) (n = 3), and high-resolution 3D surface scanning (n = 1). Stereomicroscopy (Opmi Pico, Carl Zeiss Surgical, Oberkochen, Germany) with a digital camera (Canon EOS 500D, Tokyo, Japan) was used to document active blade length, number and density of spirals (spirals/mm), and visible surface defects. SEM (Hitachi S-2400, Hitachi, Tokyo, Japan) enabled evaluation of microstructural defects, blade geometry, tip design, and surface finishing features. Three-dimensional characterization was performed with an optical 3D scanner (ATOS Q, GOM, Braunschweig, Germany) after application of a thin (~2 µm) anti-reflective coating. Each instrument was scanned over 360° from 24 orientations at a working distance of 290 mm (measuring field: 40 × 40 × 40 mm). Surface data were converted into an unmodified triangulated STL mesh and analyzed in Geomagic Control X (3D Systems, Cary, NC, USA) to measure active blade volume and surface area, axial perimeter, cross-sectional area, long axis, and core radius at 1 mm intervals from D0 to D16, as well as cross-sectional design along the entire blade length.

### 2.3. Metallurgy

Metallurgical characterization was performed using energy-dispersive X-ray spectroscopy (EDS) (n = 2) and differential scanning calorimetry (DSC) (n = 1). For EDS, three instruments per group were analyzed using a scanning electron microscope (DSM-962, Carl Zeiss, Jena, Germany) equipped with an Inca X-act detector (Oxford Instruments, Abingdon, UK) at 20 kV and 3.1 nA, with a 25 mm working distance after a 10 min vacuum. Spectra were acquired for 60 s from a 400 × 400 µm mid-blade area, and semi-quantitative elemental composition was determined using ZAF-corrected analysis (Microanalysis Suite v.4.14). DSC analysis was conducted in accordance with ASTM F2004–17 [11] using a DSC 5+ system (Mettler Toledo, Columbus, OH, USA). One fragment per group (4–5 mm) from the mid-blade was chemically etched, placed in an aluminum pan, and thermally cycled for 1 h and 40 min under nitrogen atmosphere. The cycle consisted of a 5 min stabilization at room temperature, followed by heating to 150 °C at 10 °C/min with a 2 min isothermal hold. This was followed by cooling to −150 °C at the same rate with another 2 min stabilization, then reheating to 150 °C at 10 °C/min with a further 2 min isothermal step, and finally gradual cooling back to room temperature with a 2 min stabilization. DSC curves were created and analyzed using the STARe Eval (Mettler Toledo). Phase transformation temperatures were estimated using the tangent method.

### 2.4. Mechanical Performance

Six mechanical performance parameters (time to fracture, maximum torque, maximum rotation angle, bending strength, buckling strength, and cutting efficiency) were evaluated, and the sample size was calculated based on the highest differences between systems from the initial set of six pilot tests, assuming an alpha error of 0.05 and 95% power (G*Power 3.1 for Macintosh; Heinrich Heine, Universität Düsseldorf, Germany). Effect sizes were 6.01 for time to fracture (M20 vs. M25), 3.12 for maximum torque (M20 vs. M25), 2.40 for maximum rotation angle (M25 vs. R25 Blue), 9.01 for bending strength (M20 vs. R25 Blue), 3.38 for buckling strength (M25 vs. R25 Blue), and 0.90 for cutting efficiency (M20 vs. M25). The calculated required sample sizes were 2, 2, 3, 2, 2, and 8, respectively. To ensure robust comparisons across all parameters, a uniform sample size of 10 instruments per group was adopted, providing adequate statistical power and methodological consistency.

The cyclic fatigue test (n = 10) was conducted at 35 ± 1 °C using a handpiece powered by a torque-controlled motor (X-Smart Pro+; Dentsply DeTrey GmbH, Ballaigues, Switzerland) set to the RECIPROC MINIMA or RECIPROC BLUE mode. Instruments were operated statically in a stainless-steel curved tube (19 mm length, 6 mm radius, 86° curvature), and time to fracture was recorded in seconds using a digital chronometer based on visual and auditory detection. Torsional strength (n = 10) [12] was evaluated by measuring maximum torque (N·cm) and angle of rotation (°). Instruments were mounted straight in a torsiometer (TT100; Odeme Dental Research, Luzerna, Brazil) with the apical 3 mm clamped and rotated counterclockwise at 2 rpm until fracture, with torque and rotation angle recorded using dedicated software (Odeme Analysis TT100, Luzerna, Santa Catarina, Brazil).

Bending resistance (n = 10) was evaluated by measuring maximum bending load (gf) [12]. Instruments were fixed at a 45° angle, with the apical 3 mm attached to a wire connected to a universal testing machine, and a 20 N load was applied at 15 mm/min up to a 45° displacement, with the maximum load recorded in gram-force. Buckling resistance (n = 10) was evaluated using a universal testing machine (DL-200 MF; EMIC, São José dos Pinhais, Brazil) with a 1 kN load cell, with instruments positioned vertically and stabilized at the tip [13]. A compressive load was applied axially at 1 mm/min until a 1 mm lateral displacement occurred, and the maximum buckling load was recorded in gram-force. Cutting ability (n = 10) was evaluated using a custom apparatus (Odeme OD 127; Odeme Dental Research) coupling an endodontic motor to a 500 N load cell of a universal testing machine (DL-200 MF). Instruments were operated in RECIPROC MINIMA or RECIPROC BLUE mode and introduced into a simulated straight canal (size 15/0.02) in a bone block model (PCF 10; Sawbones, Vashon, WA, USA) under a 10 gf preload. The instrument advanced 3 mm into the canal, then moved backward 2 mm, advancing 1 mm per cycle, repeated to a total depth of 10 mm. The maximum axial force recorded was used as an inverse measure of cutting efficiency [14].

### 2.5. Computational Fluid Dynamics

**Figure 1:**
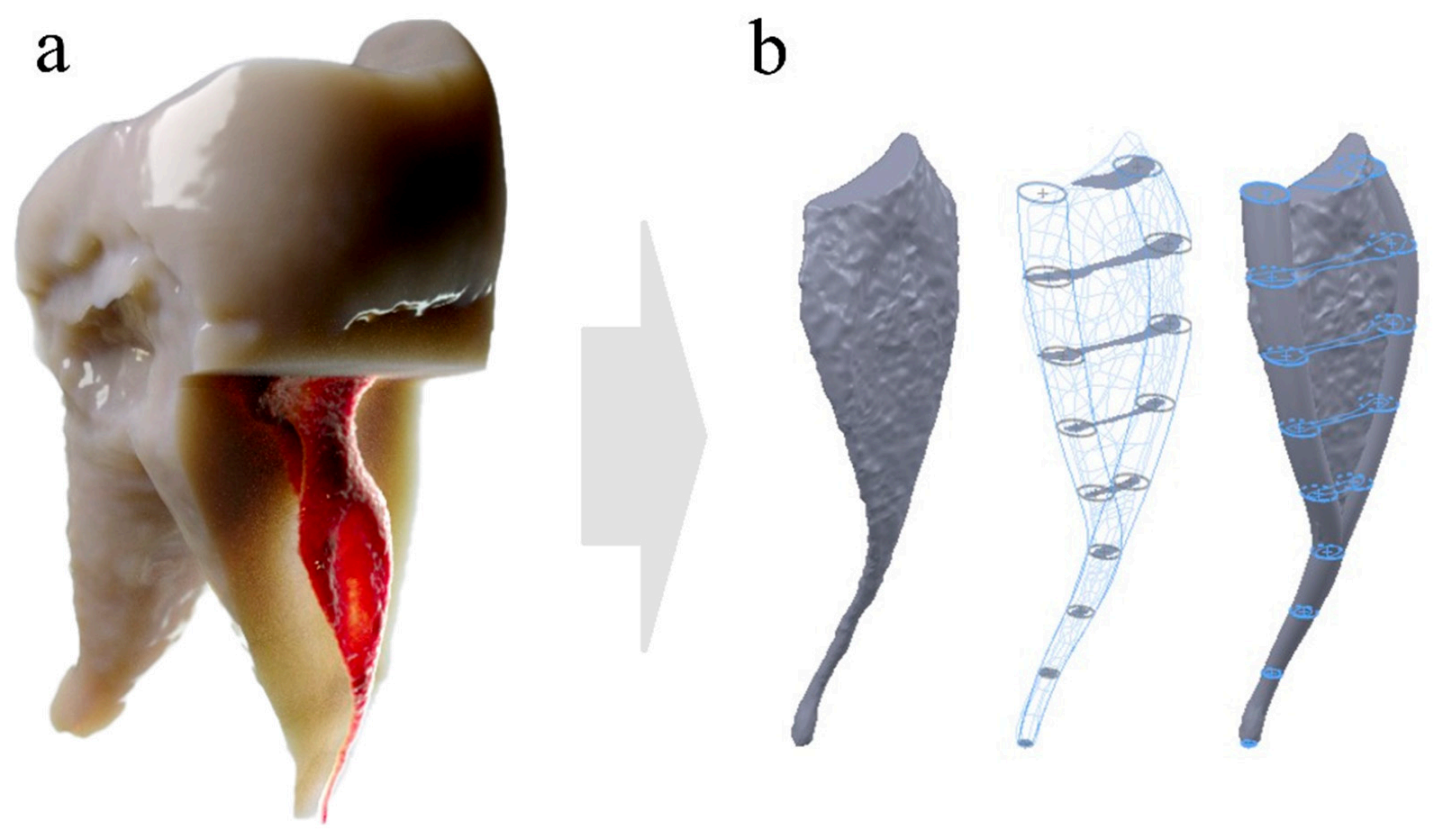
Root canal anatomy and workflow. (**a**) 3D model of a mandibular molar with Vertucci type I mesial root oval canal created from micro-CT scanning. (**b**) From left to right: 3D reconstruction of the mesial canals with axial planes every millimeter from the apical foramen, where circular profiles were created along the root canal’s long axis, based on 3D scanning measurements of real instruments; these profiles were used to generate frustums representing each instrument’s geometry, which were then added to the original anatomy to simulate the postpreparation root canal shape.

After ethics formal approval (Protocol CEBD202502), a mesial root of a mandibular molar with a severely curved (28°) Vertucci Type I oval canal was selected. The tooth was scanned by micro-CT (SkyScan 1275; Bruker-microCT, Kontich, Belgium) at 19.61 µm voxel size, and the images were reconstructed to generate an STL model of the root canal system. In SolidWorks 2017 (Dassault Systèmes, Paris, France), for each instrument group, the prepared canal geometry was reproduced by incorporating two consecutive 9 mm frustums, generated from the 3D surface-scanning measurements of the corresponding instrument (see “*Geometric design*” results), into the anatomical model (Figure 1). Based on SEM measurements (Vega3-LMU; TESCAN, Kohoutovice, Czech Republic) of commercial 30G open-ended (NaviTip; Ultradent, South Jordan, UT, USA), side-vented (Max-i-Probe; Dentsply/Tulsa Dental, York, PA, USA), and double side-vented (Irrigation Needle; Dentsply Sirona, Baillagues, Switzerland) irrigation needles, models with an outer diameter of 320 µm, an inner diameter of 170 µm, and a length of 25 mm were created and positioned in the lingual pathway of the canal with their tips located 3 mm short of the working length.

The assemblies were imported into ANSYS 2019 R2 (ANSYS, Canonsburg, PA, USA), where nine flow domains corresponding to each instrument–needle combination were created and discretized with unstructured tetrahedral meshes using ANSYS Workbench. Near-wall prism layers and local refinement were applied, and mesh-independence testing yielded final meshes of approximately 3.9–4.2 million elements. Flow simulations were performed in ANSYS Fluent using a three-dimensional, incompressible, pressure-based transient solver, assuming laminar flow without turbulence modeling [15,16]. A velocity inlet with a plug profile corresponding to 0.1 mL/s was imposed at the needle, while atmospheric pressure was set at the outlets. Canal walls were defined as rigid, impermeable, and no-slip. For the computational fluid dynamics (CFD) simulations, 2.5% NaOCl was modeled as an incompressible Newtonian fluid (ρ = 1060 kg/m^3^; μ = 1.073 × 10^−3^ Pa·s) [17]. Pressure–velocity coupling used the SIMPLE algorithm, with second-order spatial discretization (second-order upwind for momentum) and a bounded second-order implicit time scheme (Δt = 10 µs), simulating 1 s of real flow time based on prior convergence analyses [16,18]. Simulations started from rest (u = 0, *p* = 0) and were considered converged at residuals ≤0.001. Postprocessing of flow patterns, apical pressure, and wall shear stress was performed in the CFD-Post module. 

### 2.6. Statistical Analysis

Data normality was assessed separately for each experimental group using the Shapiro–Wilk test, and homogeneity of variance was verified with Levene’s test before applying one-way ANOVA. The statistical test was then selected according to the data distribution. Normally distributed data are presented as mean ± standard deviation and non-normal data as median with interquartile range. Time to fracture, maximum bending load, buckling strength, and cutting ability were analyzed by one-way ANOVA with Tukey post hoc tests, while maximum torque and angle of rotation were compared using the Kruskal–Wallis test with Student–Newman–Keuls pairwise comparisons. Statistical significance was set at 5% (SPSS v22.0; SPSS Inc., Chicago, IL, USA).

## 3. Results

### 3.1. Geometric Design

**Figure 2:**
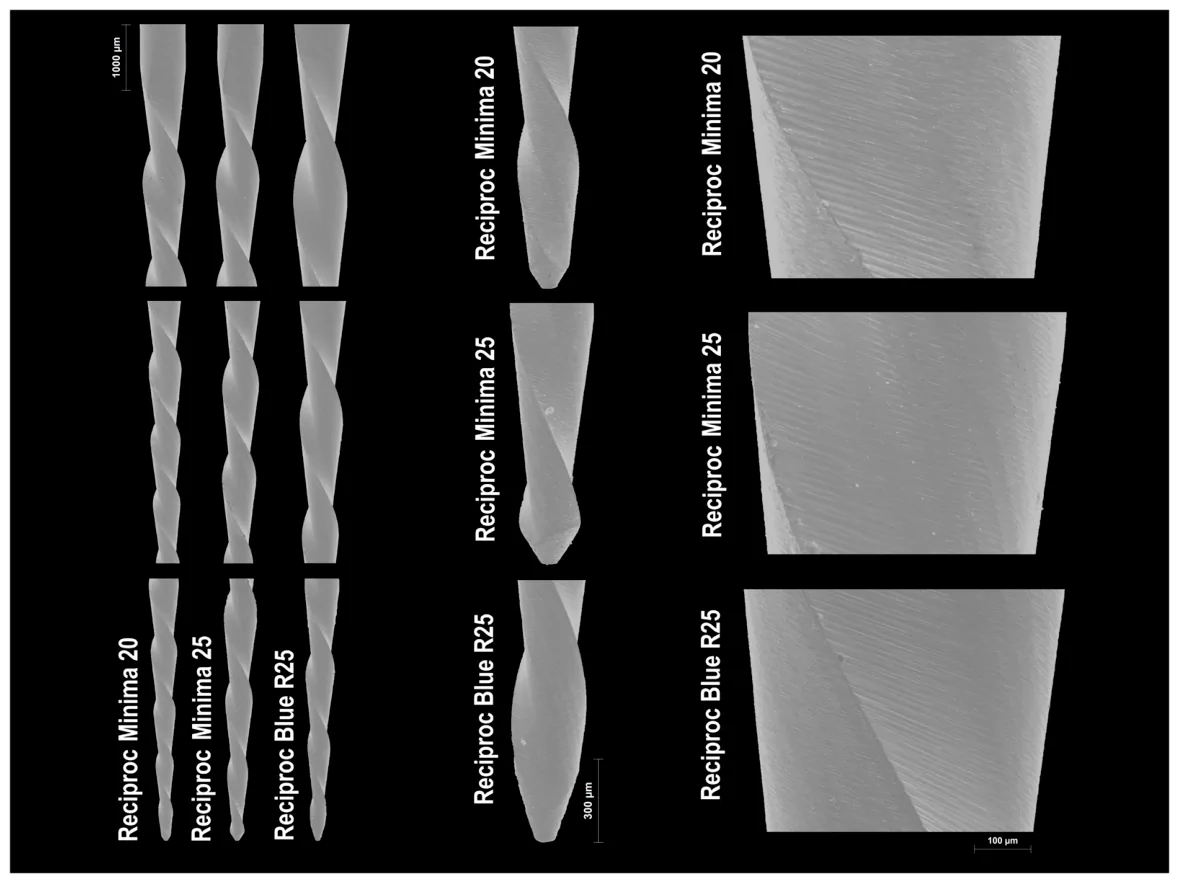
Representative SEM images. All instruments showed comparable blade morphology, characterized by parallel machining striations. The tips were non-cutting, however, the distance from the tip to D0 increased progressively, being shortest in RC Minima 20 and greatest in RC Blue R25.

**Figure 3:**
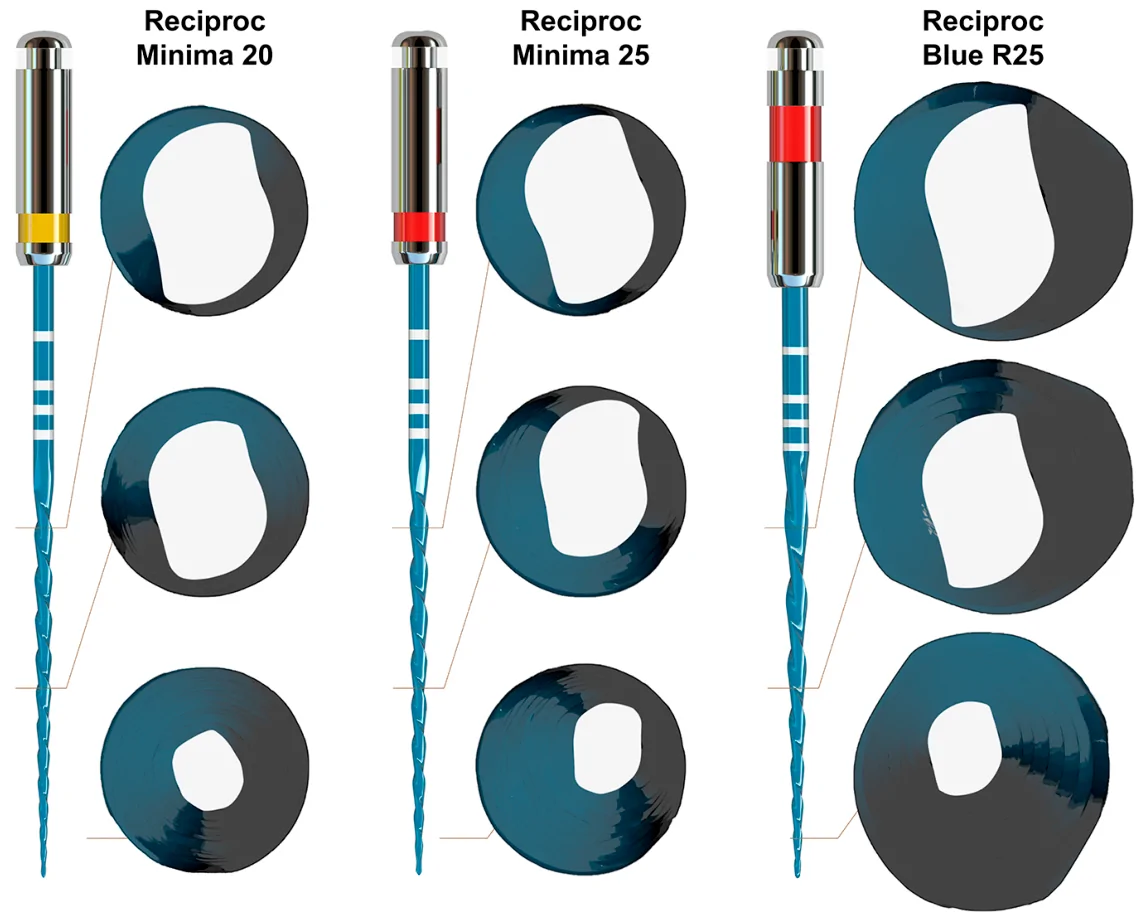
The 3D surface scanning analysis showed an S-shaped cross-section across all three groups.

Stereomicroscopy revealed differences in blade length and spiral density, with M20 showing the highest number of spirals per millimeter (0.65) and R25 Blue the lowest (0.44) (Table 1), while SEM analysis showed similar blade geometry and surface finishing, characterized by parallel machining marks and non-active tips in all instruments, although the distance from the tip to D0 increased from M20 to R25 Blue (Figure 2 and Table 2). Three-dimensional surface scanning demonstrated the lowest active blade volume and surface area in M20 (3.27 mm^3^; 26.28 mm^2^) and the highest in R25 Blue (4.35 mm^3^; 31.51 mm^2^), with M25 presenting intermediate values. Axial analysis across 17 levels confirmed an S-shaped cross-section and progressively larger dimensions from M20 to R25 Blue along the entire blade length (Table 2 and Figure 3).

**Figure 4:**
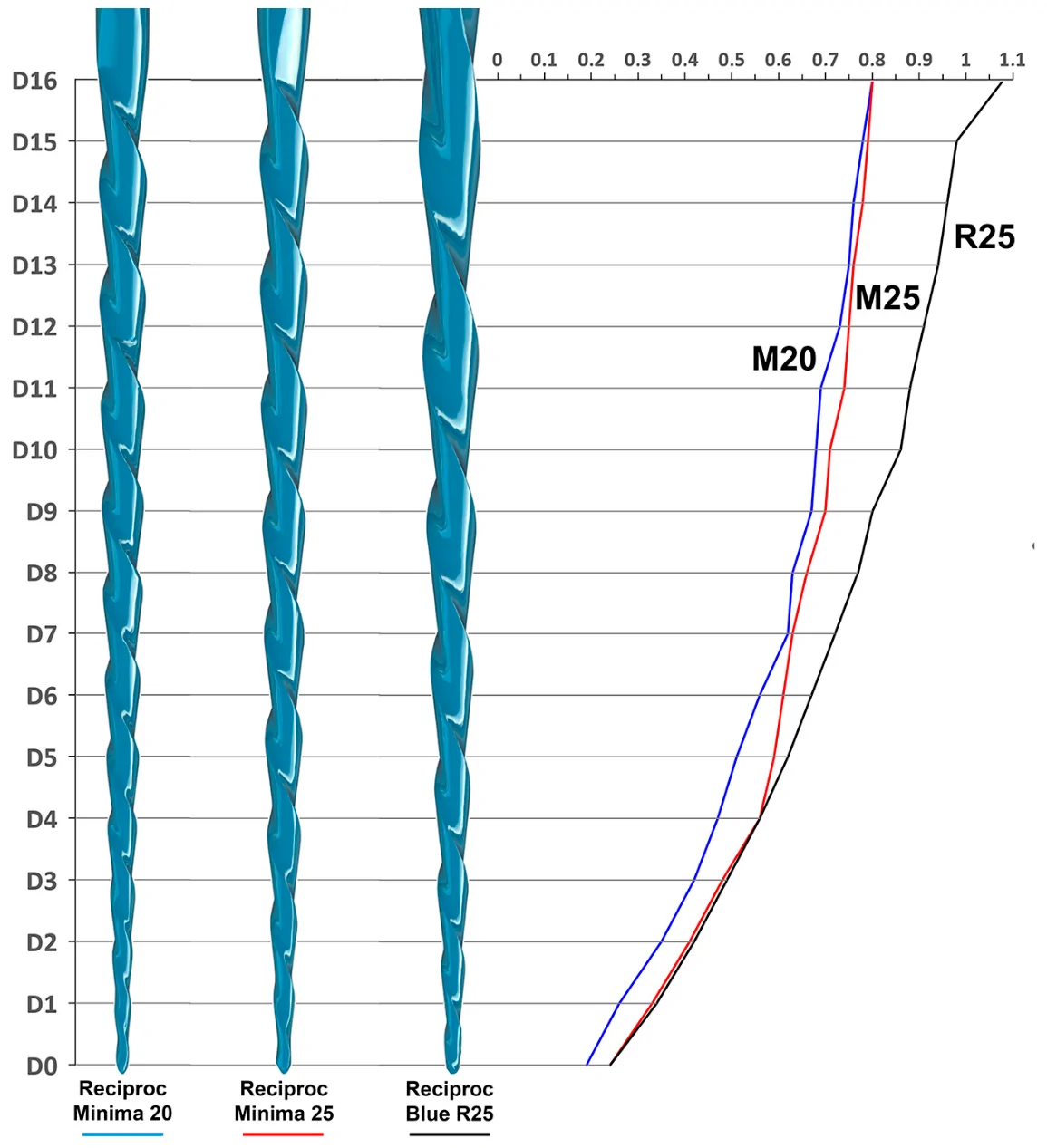
Axial analysis from D0 to D16 demonstrated a consistent increase in instrument dimensions from Reciproc Minima 20 to Reciproc Blue R25 throughout the entire active blade length.

Table 2 shows that the core radius remains broadly comparable among the three instruments along the active blade with differences between smaller and larger sizes of just 20 to 30 µm. In the long axis, R25 Blue and M25 share similar apical tip dimensions at least until D4 (0.56 mm for both), whereas M20 remains smaller (0.47 mm). From D5 onward, M25 exhibits a progressive correction, approaching M20 by around D13 (0.76 mm vs. 0.75 mm), while R25 Blue remains larger (0.94 mm) (Figure 4), a trend mirrored, although less evidently, in perimeter and area dimensions (Table 2).

### 3.2. Metallurgy

**Figure 5:**
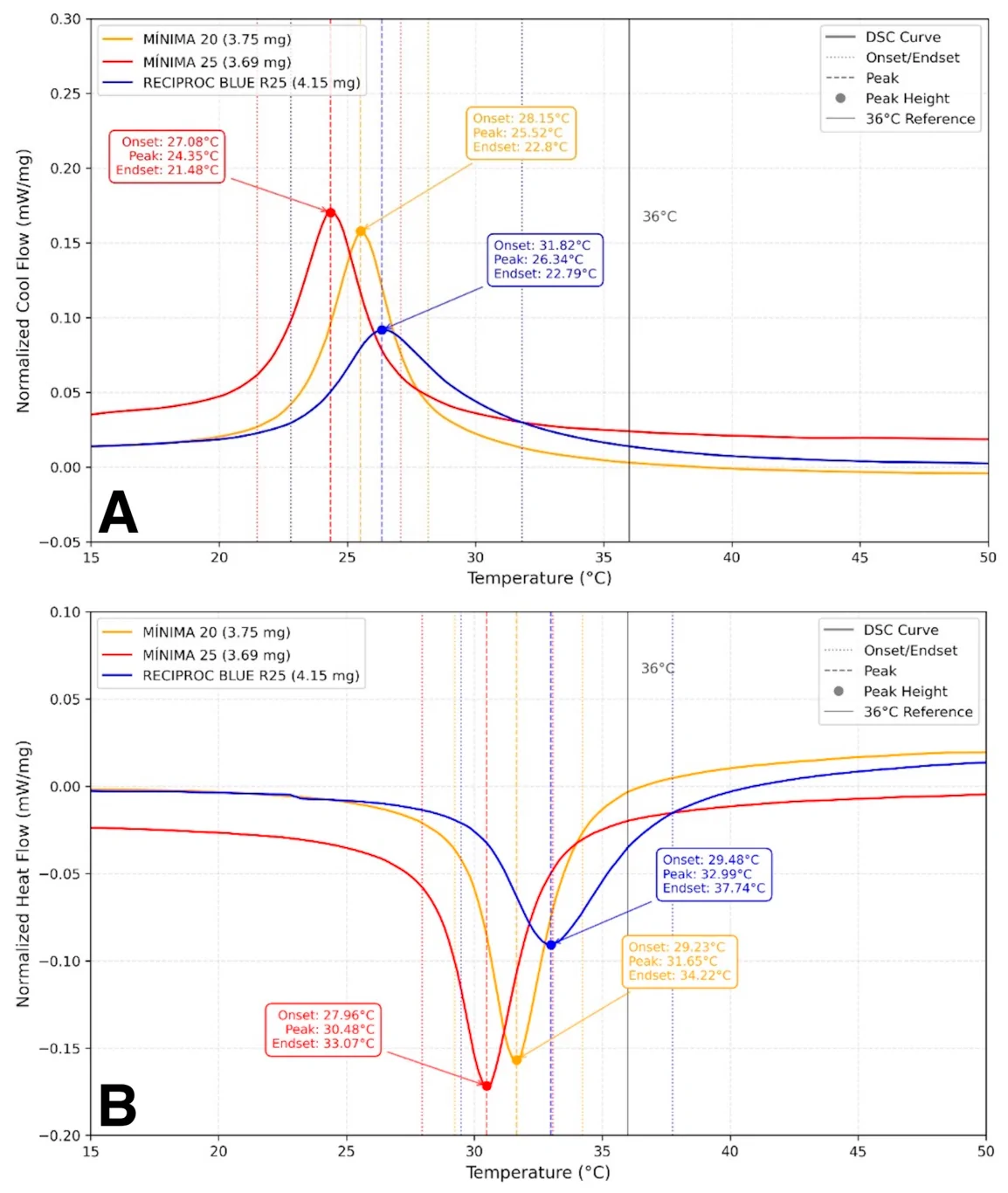
Differential scanning calorimetry (DSC) results. Cooling curves (upper panel (**A**)) are read from right to left, and heating curves (lower panel (**B**)) from left to right. R-phase transformation occurred between 20 °C and 36 °C, with Rs at 27–31 °C and Rf at 21–22 °C, while austenite transformation started at 27–29 °C (As) and finished at 33–37 °C (Af). Fragment weights were comparable (3.69–4.15 mg). Smaller fragments exhibited higher DSC peaks and slightly lower transformation temperatures, whereas heavier fragments showed lower peak intensity and slightly higher temperatures, with all values remaining within the same magnitude range.

EDS confirmed that all Reciproc instruments were composed of near-equiatomic NiTi alloy, with no detectable traces of other metallic elements (Table 1). DSC showed comparable phase transformation temperatures among groups, indicating similar heat treatments, with R-phase transformations between 20 °C and 36 °C. Specifically, Rs ranged from 27 °C to 31 °C, Rf from 21 °C to 22 °C, As from 27 °C to 29 °C, and Af from 33 °C to 37 °C (Figure 5). Fragment weights were similar (3.69–4.15 mg), with lighter fragments exhibiting higher DSC peaks and slightly lower transformation temperatures and heavier fragments showing lower peaks and slightly higher temperatures, all within comparable ranges.

### 3.3. Mechanical Performance

M20 showed the highest cyclic fatigue resistance (348 ± 15 s), followed by R25 Blue (257 ± 14 s), while M25 had the lowest values (240 ± 12 s) (*p* < 0.0001). Maximum torque was higher for R25 Blue and M25 (1.6 N·cm) than for M20 (1.2 N·cm) (*p* < 0.0001), while angular deflection was greatest in M25 (577°), followed by M20 (496°) and R25 Blue (443°) (*p* < 0.0001). M25 showed the best cutting performance (141 gf), M20 the lowest (161 gf), and R25 Blue intermediate values (154 gf) (*p* = 0.02). In flexibility tests, M20 and M25 (152–157 gf) were significantly more flexible than R25 Blue (295 gf), whereas buckling resistance was highest for R25 Blue (324 gf), intermediate for M25 (253 gf), and lowest for M20 (181 gf) (*p* < 0.0001) (Table 3).

### 3.4. Computational Fluid Dynamics

**Figure 6:**
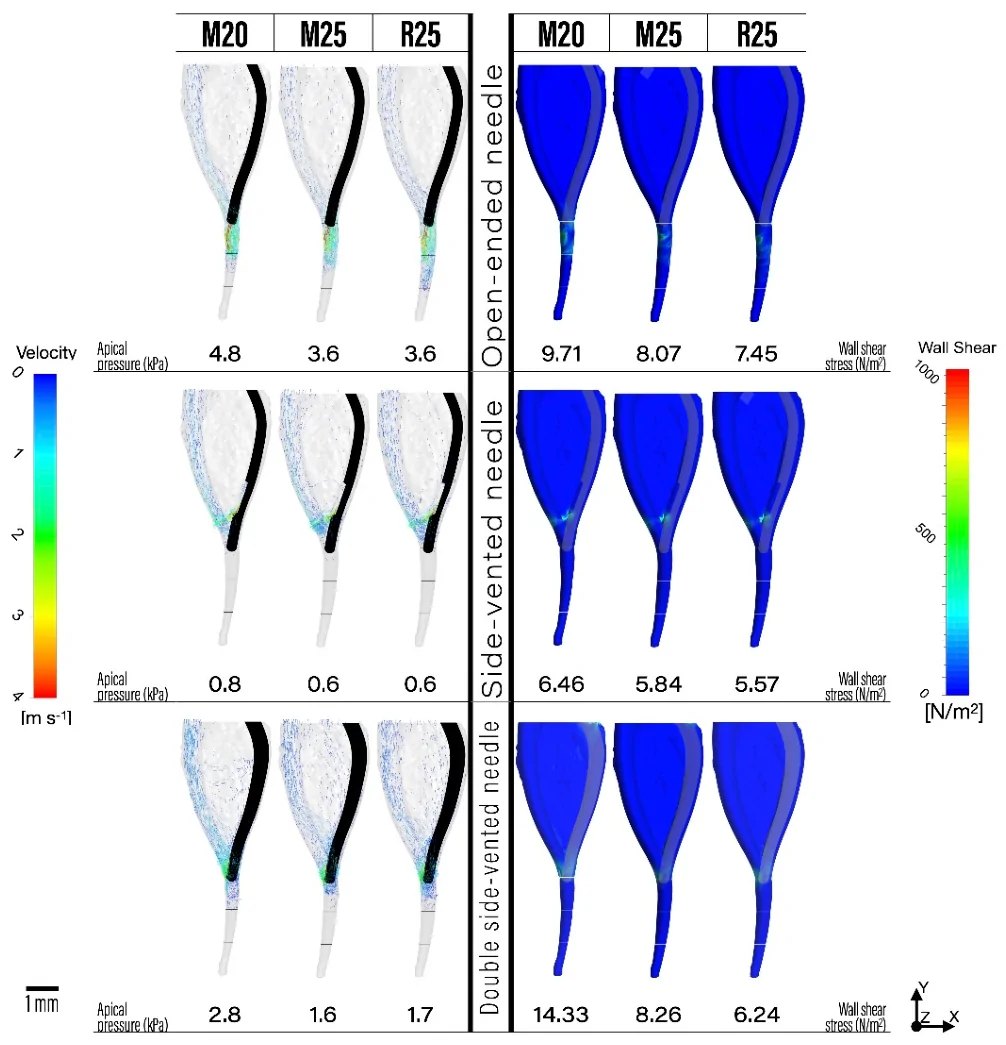
Computational fluid dynamics results. (**Left**): Velocity vectors and direction of massless particles within the root canal (at t = 1 s), colored by velocity magnitude and apical pressure (kPa) for the open-ended (**top**), side-vented (**middle**), and double side-vented (**bottom**) needles, placed 3 mm short of the working length for all root canal preparations assessed (RC Minima 20 [R20], RC Minima 25 [R25 m], RC Blue R25 [R25]). (**Right**): Contours and maximum time-averaged wall shear stress (N/m^2^) for all evaluated cases.

CFD results are shown in Figure 6. With the open-ended needle, the irrigant jet was directed apically and exited mainly through the flow pathway opposite the needle within the canal lumen, whereas the side-vented needle produced lateral flow toward the same canal without apical penetration beyond the needle tip. The double side-vented needle generated a flow pattern similar to the open-ended design but with reduced apical penetration (~1 mm vs. 1.5–2 mm). None of the needle designs delivered irrigant to the working length in canals prepared with any Reciproc instrument. Greater apical penetration was observed only in canals prepared with R25 Blue when using the open-ended needle, compared with M20 and M25. Maximum wall shear stress occurred near the outlets of side-vented needles and apical to the tip of open-ended needles, with M20 showing the highest values and R25 Blue the lowest across all needle types. Apical pressure decreased sequentially with needle design (open-ended > double side-vented > side-vented) and instrument size (M20 > M25 > R25 Blue).

## 4. Discussion

The present study provides the first comprehensive laboratory-based evaluation of the Reciproc Minima system and expands the current evidence on how conservative taper design modulates the geometric, mechanical, and irrigation-related behavior of reciprocating NiTi instruments. By combining high-resolution 3D geometric analysis, metallurgical characterization, an extensive mechanical testing protocol, and computational fluid dynamics, this investigation follows the integrative, mechanism-oriented approach adopted in a recent publication [14]. The findings demonstrate that, despite identical reciprocating kinematics, near-equiatomic NiTi composition, and comparable phase transformation behavior, Reciproc Minima instruments exhibit distinct mechanical responses and irrigation dynamics when compared with R25 Blue. Accordingly, the null hypothesis was rejected.

The metallurgical analyses confirmed that all instruments exhibited near-equiatomic NiTi composition with no detectable alloying differences (Table 1) and highly similar phase transformation temperatures, with R-phase transformation occurring around body temperature (Figure 5). Together, these findings indicate that Reciproc Minima and Reciproc Blue share the same heat-treatment concept. The minor variations observed in DSC peak height and width are more plausibly explained by differences in fragment mass and thermal contact within the DSC pan, where lighter fragments facilitate more efficient heat transfer and heavier fragments produce broader, less intense peaks, rather than by intrinsic differences in alloy microstructure, a phenomenon that falls within expected experimental variability and has been previously reported in thermal analyses of NiTi alloys [19].

From a design perspective, Reciproc Minima instruments were characterized by smaller blade volume, reduced core dimensions, and higher spiral density compared with R25 Blue (Table 1 and Table 2, Figure 2, Figure 3 and Figure 4). These features are a direct consequence of the regressive and overall lower taper of the Minima instruments and are in line with previous multimethod investigations demonstrating that taper and metal mass are dominant determinants of instrument behavior when alloy composition and kinematics are controlled [6,14]. From a clinical standpoint, such design modifications are particularly relevant within the contemporary framework of minimally invasive endodontics, in which preservation of radicular and pericervical dentin is increasingly regarded as a key determinant of long-term tooth survival [2,3]. Importantly, these geometric differences were observed in the absence of metallurgical variability, as confirmed by EDS (Table 1) and DSC analyses (Figure 5), thereby reinforcing that the mechanical performance variations among the instruments were driven by geometric design rather than material or kinematic differences. These findings are consistent with the multimethod analysis by Silva et al. [14], who demonstrated that low-taper reciprocating instruments generally exhibit reduced core dimensions, increased flexibility, and improved cyclic fatigue resistance compared with larger-taper systems, while maintaining similar metallurgical characteristics. The present study extends those observations by specifically evaluating the newly introduced Reciproc Minima system and demonstrating that, even within the same reciprocating platform and heat treatment, taper reductions together with differences in core dimensions, blade volume, spiral density, and blade length are sufficient to produce meaningful differences in both mechanical behavior and irrigation dynamics. Although these findings support the mechanical rationale for conservative instrumentation, the present study did not evaluate canal shaping outcomes, dentin preservation, cleaning efficacy, or clinical performance. Therefore, any potential clinical advantages associated with the reduced taper of Reciproc Minima remain hypothetical and require confirmation in experimental and clinical studies.

In the cyclic fatigue resistance test, M20 exhibited the longest time to fracture, followed by R25 Blue and M25 (Table 3). The superior fatigue resistance of M20 is consistent with its smaller core diameter (Table 2) and higher flexibility (Table 3), which reduce tensile–compressive stress accumulation at the point of maximum curvature. This finding aligns with extensive literature showing that reduced metal mass and enhanced flexibility are key contributors to improved fatigue life in curved canals [6,14,20]. In contrast, M25, despite being more flexible than R25 Blue, showed the lowest fatigue resistance (Table 3). This apparently counterintuitive finding highlights the multifactorial nature of cyclic fatigue behavior and suggests that, although a thinner core reduces overall bending stiffness, it also reduces the cross-section at the region of maximum curvature, so that once microcracks initiate, their coalescence and catastrophic failure may occur earlier. Importantly, the mean fatigue resistance of M25 remained within the same order of magnitude as that of R25 Blue, suggesting that this difference is unlikely to be clinically limiting when the instrument is used within its intended indications.

Torsional testing further illustrated the complex interaction between taper, flexibility, and mechanical performance (Table 3). M25 demonstrated the greatest angular deflection before fracture while maintaining maximum torque values comparable to R25 Blue. This combination is noteworthy, as increased angular deflection reflects enhanced ductility and a greater capacity for plastic deformation prior to failure, which may reduce the risk of sudden fracture under clinical torsional overload [21]. The preservation of torque resistance is plausibly explained by the similar apical dimensions of M25 and R25 Blue at the D3 level (Table 2 and Figure 4), where torsional testing is standardized according to ISO specifications [12]. Thus, while taper reduction increased flexibility and angular deflection, preservation of apical mass allowed torsional strength to be maintained, demonstrating that conservative taper designs can achieve a favorable mechanical balance when appropriately engineered.

Buckling resistance followed a predictable geometric pattern, with R25 Blue exhibiting the highest values, M25 intermediate performance, and M20 the lowest resistance (Table 3). These findings are consistent with previous studies showing that buckling strength is directly related to instrument stiffness, taper, and overall metallic mass [10,13]. From a clinical standpoint, higher buckling resistance facilitates instrument penetration in constricted or calcified canals, while lower buckling resistance is associated with enhanced flexibility [22]. However, the clinical relevance of buckling resistance is instrument-size dependent. Higher buckling resistance is particularly important for larger instruments, such as R25 Blue, which may be subjected to higher axial loads when negotiating narrow, constricted, or calcified canals. In contrast, buckling resistance becomes less critical for smaller instruments, such as M25 and especially M20, whose reduced dimensions allow canal negotiation with minimal or no applied pressure. Therefore, the reduced buckling resistance of M20 should be interpreted as an inherent trade-off associated with designs prioritizing conservative shaping rather than as an isolated limitation.

Cutting efficiency was comparable among the instruments, with M25 and R25 Blue showing similar performance and M20 exhibiting slightly lower efficiency (Table 3). These results support the concept that axial cutting is not determined by a single mechanical parameter, such as buckling resistance, but by the interaction between geometry, flexibility, and contact mechanics [4,10]. Despite its reduced taper, M25 appears to achieve efficient dentin removal through improved canal negotiation and reduced binding, whereas the lower cutting efficiency of M20 likely reflects its minimal core mass and reduced contact pressure. From a clinical perspective, the mechanical profile of M20 may be advantageous in complex anatomies, where controlled cutting and improved centering ability are preferable to aggressive dentin removal.

The CFD analysis provided additional insights into how geometric reduction influences irrigation dynamics (Figure 6). The simulations were intentionally performed under standardized conditions using a single anatomical model, a single canal curvature, a fixed needle insertion depth (3 mm short of the working length), a constant flow rate (0.1 mL/s), a Newtonian model of 2.5% NaOCl, rigid and impermeable canal walls, and static needle positioning without dynamic movement or activation. In agreement with previous experimental studies [1,9,15], needle design was the primary determinant of irrigant flow pattern and apical penetration. None of the instrument–needle combinations enabled irrigant delivery to the working length, which is consistent with reports showing limited apical penetration in canals prepared to similar apical sizes when the needle is positioned safely short of the working length [23,24]. Increasing instrument diameter and taper resulted in slightly greater apical penetration and lower apical pressure, particularly for R25 Blue used with an open-ended needle. Reduced apical pressure may be interpreted as a safety advantage, as it is associated with a lower risk of irrigant extrusion beyond the apical foramen.

Conversely, M20 generated the highest wall shear stress values along the canal walls (Figure 6). Higher wall shear stress has been associated with enhanced mechanical disruption of biofilms and improved debris removal during syringe irrigation [1,9]. However, this benefit was accompanied by higher apical pressure values, reflecting the reduced canal volume available for irrigant flow. These findings are consistent with previous CFD studies demonstrating that larger preparations favor irrigant penetration and safety, whereas smaller preparations increase wall shear stress at the expense of apical pressure control [1,9,16,23,24]. Therefore, the irrigation results highlight a clinically relevant trade-off between conservative shaping and irrigant delivery efficiency.

From a clinical perspective, the irrigation dynamics observed in this study suggest that instrument geometry may influence the balance between conservative canal preparation and irrigation effectiveness. The higher wall shear stress generated in canals prepared with the lower-taper Minima instruments may theoretically improve irrigant replacement and enhance biofilm disruption along the canal walls, whereas the lower apical pressures observed after preparation with Reciproc Blue R25 may reduce the potential risk of irrigant extrusion beyond the apical foramen. Although none of the evaluated needle designs allowed irrigant to reach the working length, the greater apical penetration observed after preparation with Reciproc Blue R25 suggests that its larger preparation may facilitate irrigant exchange in the apical third. Consequently, clinicians should balance the benefits of conservative dentin preservation offered by low-taper instruments against the potentially improved irrigant exchange associated with larger preparations, while considering the anatomical characteristics and biological objectives of each individual case. Because these observations were derived from computational fluid dynamics simulations, they should be interpreted as mechanistic predictions rather than direct clinical evidence, and further experimental and clinical studies are needed to determine their clinical significance.

Several strengths of this study should be highlighted. First, this is the first investigation to characterize the Reciproc Minima system, providing foundational data for future experimental and clinical research. Second, by standardizing alloy composition, heat treatment, and kinematics, the study isolated the influence of geometric design with a high degree of internal validity. Third, the multimethod approach, combining mechanical testing, advanced geometric analysis, and CFD, aligns with contemporary recommendations for comprehensive evaluation of NiTi instruments and allows for more nuanced interpretation of performance trade-offs [4,10,25]. A static cyclic fatigue model was used because it provides a highly standardized and reproducible method for comparing the intrinsic fatigue resistance of different instruments under identical testing conditions, although it does not fully reproduce the dynamic axial movements occurring during clinical instrumentation [26]. The CFD analysis [27] was performed using a single anatomically representative root canal model under standardized irrigation conditions to isolate the influence of instrument geometry, therefore, the findings should be interpreted with caution when extrapolating them to different anatomical configurations or clinical scenarios. Nevertheless, the present study has several limitations. The computational fluid dynamics analysis was based on a single micro-CT-derived root canal anatomy with one canal configuration, one degree of curvature, a fixed needle insertion depth and irrigant flow rate, while assuming rigid and impermeable canal walls, Newtonian fluid behavior, and static syringe irrigation without needle movement or irrigant activation. Consequently, the simulated flow patterns represent mechanistic predictions under standardized conditions and may not fully reproduce the variability encountered in clinical practice. Furthermore, the study did not evaluate biological or clinical outcomes, such as dentin removal, biofilm disruption, irrigant extrusion, postoperative response, or treatment success, nor were the CFD predictions experimentally validated. Finally, although alloy composition and reciprocating kinematics were controlled, the tested instruments differed in several geometric characteristics, including taper, core dimensions, blade volume, spiral density, blade length, and tip size. Therefore, the observed differences should be interpreted as the combined effect of the overall instrument geometry rather than the isolated influence of taper. Future experimental and clinical studies incorporating different anatomical configurations and biological validation are warranted to confirm the clinical relevance of these findings.

From a clinical perspective, the present findings suggest that Reciproc Minima instruments may be particularly advantageous in cases requiring enhanced flexibility, dentin preservation, and improved resistance to cyclic fatigue, such as narrow roots and severely curved canals. These benefits appear to be achieved without substantial compromise in torsional strength or cutting efficiency, especially for M25. However, clinicians should remain aware of the lower buckling resistance associated with smaller tapers and adapt their instrumentation strategy accordingly. Future studies should investigate shaping outcomes, debris accumulation, and clinical performance, as well as the interaction of Reciproc Minima instruments with activated irrigation protocols and their potential impact on long-term tooth survival.

## 5. Conclusions

Although Reciproc Minima and Reciproc Blue R25 exhibited similar metallurgical characteristics, differences in geometric design were associated with distinct mechanical properties and irrigation fluid dynamics. These differences suggest that instrument selection may be tailored according to the desired balance between conservative dentin preservation, mechanical performance, and irrigation effectiveness during root canal treatment.

## Figures and Tables

**Table 1:** Design characteristics and elements ratio of the tested instruments.

Instrument	Lot Number	Ni/Ti Ratio	Design Characteristics				
			Blade Length	Spirals	Spirals per mm	Volume (mm^3^)	Surface Area (mm^2^)
Reciproc Minima 20	1891481	1.037	17 mm	11	0.65	3.27	26.28
Reciproc Minima 25	1891482	1.021	17 mm	9	0.52	3.38	27.74
Reciproc Blue R25	462818	1.063	18 mm	8	0.44	4.35	31.51

**Table 2:** Measurements of the active blade dimensions of the tested instruments from D0 to D16.

Instruments	D0	D1	D2	D3	D4	D5	D6	D7	D8	D9	D10	D11	D12	D13	D14	D15	D16
Perimeter (mm)																	
Reciproc Minima 20	0.49	0.78	0.99	1.20	1.35	1.45	1.53	1.67	1.78	1.83	1.88	1.92	1.97	2.00	2.03	2.08	2.29
Reciproc Minima 25	0.65	0.91	1.13	1.33	1.47	1.56	1.64	1.70	1.78	1.85	1.91	1.96	1.98	2.01	2.03	2.06	2.40
Reciproc Blue R25	0.67	0.93	1.17	1.35	1.52	1.67	1.79	1.92	2.04	2.13	2.24	2.29	2.34	2.38	2.42	2.46	2.81
Area (mm^2^)																	
Reciproc Minima 20	0.02	0.04	0.07	0.11	0.13	0.15	0.17	0.20	0.23	0.24	0.25	0.26	0.27	0.28	0.29	0.31	0.39
Reciproc Minima 25	0.03	0.06	0.09	0.13	0.15	0.17	0.19	0.20	0.22	0.24	0.26	0.27	0.27	0.28	0.29	0.30	0.43
Reciproc Blue R25	0.03	0.06	0.10	0.13	0.17	0.20	0.23	0.26	0.29	0.32	0.34	0.36	0.36	0.37	0.38	0.39	0.58
Long axis (mm)																	
Reciproc Minima 20	0.19	0.26	0.35	0.42	0.47	0.51	0.56	0.62	0.63	0.67	0.68	0.69	0.73	0.75	0.76	0.78	0.80
Reciproc Minima 25	0.24	0.33	0.41	0.48	0.56	0.59	0.61	0.63	0.66	0.70	0.71	0.74	0.75	0.76	0.78	0.79	0.80
Reciproc Blue R25	0.24	0.34	0.42	0.49	0.56	0.62	0.67	0.72	0.77	0.80	0.86	0.88	0.91	0.94	0.96	0.98	1.08
Core radius (mm)																	
Reciproc Minima 20	0.06	0.10	0.12	0.15	0.17	0.18	0.19	0.21	0.22	0.22	0.23	0.23	0.23	0.23	0.24	0.24	0.28
Reciproc Minima 25	0.08	0.11	0.14	0.16	0.17	0.18	0.19	0.20	0.21	0.22	0.22	0.22	0.22	0.23	0.23	0.23	0.30
Reciproc Blue R25	0.09	0.12	0.15	0.17	0.19	0.20	0.21	0.22	0.24	0.24	0.25	0.25	0.25	0.25	0.25	0.25	0.36

**Table 3:** Mean (standard deviation) or median [interquartile range] mechanical results.

	Reciproc Minima 20	Reciproc Minima 25	Reciproc Blue R25	*p*-Value
Time to fracture (sec)	348 ± 15 ^A^	240 ± 12 ^C^	257 ± 14 ^B^	<0.0001
Maximum torque (N·cm)	1.2 [1.1–1.2] ^A^	1.6 [1.5–1.7] ^B^	1.6 [1.5–1.7] ^B^	<0.0001
Angle of rotation (°)	496 [478–510] ^B^	577 [561–589] ^A^	443 [427–452] ^C^	<0.0001
Bending load (gf)	152 ± 9 ^A^	157 ± 8 ^A^	295 ± 22 ^B^	<0.0001
Buckling strength (gf)	181 ± 16 ^A^	253 ± 6 ^B^	324 ± 25 ^C^	<0.0001
Cutting ability (gf)	161 ± 12 ^A^	141 ± 16 ^B^	154 ± 16 ^AB^	0.02

Different superscript letters in the same row represent statistically significant differences (*p* < 0.05).

## Data Availability

Data are contained within the article.

## References

[1] Boutsioukis C., Arias-Moliz M.T. (2022). Present status and future directions—Irrigants and irrigation methods. Int. Endod. J..

[2] Patel S., Bhuva B., Bose R. (2022). Present status and future directions: Vertical root fractures in root filled teeth. Int. Endod. J..

[3] Silva E.J.N.L., De-Deus G., Souza E.M., Belladonna F.G., Cavalcante D.M., Simoes-Carvalho M., Versiani M.A. (2022). Present status and future directions—Minimal endodontic access cavities. Int. Endod. J..

[4] Martins J.N.R., Silva E.J.N.L., Marques D., Baruwa A.O., Caramês J., Braz Fernandes F.M., Versiani M.A. (2023). Unveiling the performance of nickel-titanium endodontic instruments through multimethod research: A review. App. Sci..

[5] Grande N.M., Ahmed H.M., Cohen S., Bukiet F., Plotino G. (2015). Current assessment of reciprocation in endodontic preparation: A comprehensive review-part I: Historic perspectives and current applications. J. Endod..

[6] Martins J.N.R., Silva E.J.N.L., Rito Pereira M., Marques D., Pinto R., Baruwa A.O., Martins R.F., Braz Fernandes F.M., Versiani M.A. (2025). Characterising 12 NiTi reciprocating systems: An integrative laboratorial analysis of design, mechanics, metallurgy, fluid dynamics and finite element analysis. Int. Endod. J..

[7] Dental V.D.W. (2021). Reciproc Family Product Brochure.

[8] Jamleh A. (2022). Effect of blue heat treatment on vertical force induced with endodontic files. Eur. Endod. J..

[9] Pereira T.C., Boutsioukis C., Dijkstra R.J.B., Petridis X., Versluis M., de Andrade F.B., van de Meer W.J., Sharma P.K., van der Sluis L.W.M., So M.V.R. (2021). Biofilm removal from a simulated isthmus and lateral canal during syringe irrigation at various flow rates: A combined experimental and computational fluid dynamics approach. Int. Endod. J..

[10] Schäfer E., Bürklein S., Donnermeyer D. (2022). A critical analysis of research methods and experimental models to study the physical properties of NiTi instruments and their fracture characteristics. Int. Endod. J..

[11] (2004). Standard Test Method for Transformation Temperature of Nickel-Titanium Alloys by Thermal Analysis.

[12] (2008). Dentistry—Root Canal Instruments—Part 1: General Requirements and Test Methods.

[13] Lopes H.P., Elias C.N., Mangelli M., Lopes W.S., Amaral G., Souza L.C., Siqueira J.F. (2012). Buckling resistance of pathfinding endodontic instruments. J. Endod..

[14] Silva E.J.N.L., Pena-Bengoa F., Ajuz N.C., Vieira V.T.L., Martins J.N.R., Marques D., Pinto R., Rito Pereira M., Braz-Fernandes F.M., Versiani M.A. (2024). Multimethod analysis of large- and low-tapered single file reciprocating instruments: Design, metallurgy, mechanical performance, and irrigation flow. Int. Endod. J..

[15] Boutsioukis C., Verhaagen B., Versluis M., Kastrinakis E., van der Sluis L.W. (2010). Irrigant flow in the root canal: Experimental validation of an unsteady computational fluid dynamics model using high-speed imaging. Int. Endod. J..

[16] Rito Pereira M., Silva G., Semiao V., Silverio V., Martins J.N.R., Pascoal-Faria P., Alves N., Dias J.R., Ginjeira A. (2022). Experimental validation of a computational fluid dynamics model using micro-particle image velocimetry of the irrigation flow in confluent canals. Int. Endod. J..

[17] Guerisoli D.M.Z., Silva R.S., Pécora J.D. (1998). Evaluation of some physico-chemical properties of different concentrations of sodium hypochlorite solutions. Braz. Endod. J..

[18] Wang R., Shen Y., Ma J., Huang D., Zhou X., Gao Y., Haapasalo M. (2015). Evaluation of the effect of needle position on irrigant flow in the C-shaped root canal using a computational fluid dynamics model. J. Endod..

[19] Ghanbari E., Picken S.J., van Esch J.H. (2023). Analysis of differential scanning calorimetry (DSC): Determining the transition temperatures, and enthalpy and heat capacity changes in multicomponent systems by analytical model fitting. J. Therm. Anal. Calorim..

[20] Bürklein S., Zupanc L., Donnermeyer D., Tegtmeyer K., Schäfer E. (2021). Effect of core mass and alloy on cyclic fatigue resistance of different nickel-titanium endodontic instruments in matching artificial canals. Materials.

[21] McSpadden J.T. (2007). Mastering the concepts. Mastering Endodontic Instrumentation.

[22] Elnaghy A.M., Elsaka S.E. (2015). Evaluation of the mechanical behaviour of PathFile and ProGlider pathfinding nickel-titanium rotary instruments. Int. Endod. J..

[23] Boutsioukis C., Gogos C., Verhaagen B., Versluis M., Kastrinakis E., Van der Sluis L.W. (2010). The effect of apical preparation size on irrigant flow in root canals evaluated using an unsteady computational fluid dynamics model. Int. Endod. J..

[24] Boutsioukis C., Lambrianidis T., Verhaagen B., Versluis M., Kastrinakis E., Wesselink P.R., van der Sluis L.W. (2010). The effect of needle-insertion depth on the irrigant flow in the root canal: Evaluation using an unsteady computational fluid dynamics model. J. Endod..

[25] Silva E.J.N.L., Martins J.N.R., Versiani M.A. (2026). NiTi instruments in endodontics: Contemporary concepts, principles, and practical guidance. Braz. Oral Res..

[26] Thu M., Ebihara A., Maki K., Mike N., Okiji T. (2020). Cyclic fatigue resistance of rotary and reciprocating nickel-titanium instruments subjected to static and dynamic tests. J. Endod..

[27] Boutsioukis C., Nova P.G. (2021). Syringe irrigation in minimally shaped root canals using three endodontic needles: A Computational Fluid Dynamics study. J. Endod..

